# Insecticide Resistance and Its Intensity in Populations of Malaria Vectors in Colombia

**DOI:** 10.1155/2018/9163543

**Published:** 2018-08-29

**Authors:** Lorena I. Orjuela, Juliana A. Morales, Martha L. Ahumada, Juan F. Rios, John J. González, Johana Yañez, Angelo Rosales, Diana M. Cabarcas, Juan Venegas, Maria F. Yasnot, Martha L. Quiñones

**Affiliations:** ^1^Grupo de Investigaciones Microbiológicas y Biomédicas de Córdoba-GIMBIC, Universidad de Córdoba, Monteria 230001, Colombia; ^2^Universidad de Cartagena, Facultad de Medicina, Sede Zaragocilla, Calle 30 N° 48-152, Cartagena de Indias, Bolivar, Cartagena 1300, Colombia; ^3^Grupo de Entomología, Instituto Nacional de Salud, Bogotá D.C. 110111, Colombia; ^4^Secretaria de Salud de Antioquía, Instituto Colombiano de Medicina Tropical, Medellín 050001, Colombia; ^5^Residente del Programa de Epidemiología del Campo FETP, Instituto Nacional de Salud, Bogotá D.C. 110111, Colombia; ^6^Instituto Departamental de Salud de Norte de Santander, Cúcuta 540001, Colombia; ^7^Secretaria de Salud Distrital de Buenaventura, Buenaventura 764501, Colombia; ^8^Secretaria de Salud Departamental de Córdoba, Monteria 230001, Colombia; ^9^Programa de Biología Celular y Molecular, Instituto de Ciencias Biomédicas, Santiago de Chile 8320000, Chile; ^10^Departamento de Salud Pública, Universidad Nacional, Bogotá D.C. 110111, Colombia

## Abstract

Insecticide resistance in malaria vectors threatens malaria prevention and control efforts. In Colombia the three primary vectors,* Anopheles darlingi, An. nuneztovari s.l.*, and* An. albimanus*, have reported insecticide resistance to pyrethroids, organophosphates, carbamates, and DDT; however, the insecticide resistance monitoring is not continuous, and the data on the prevalence of resistance is scarce and geographically limited. We describe the resistance levels and intensity of previously detected resistant populations among primary malaria vectors from the most endemic malaria areas in Colombia. The study was carried out in 10 localities of five states in Colombia. Bioassays were carried out following the methodology of CDC Bottle Bioassay using the discriminating concentration and in order to quantify the intensity the specimens were exposed to 2, 5, and 10X discriminating concentrations. Five insecticides were tested: deltamethrin, lambda-cyhalothrin, alpha-cypermethrin, permethrin, and DDT. The results provide evidence of low resistance intensity and resistance highly localized to pyrethroids and DDT in key malaria vectors in Colombia. This may not pose a threat to malaria control yet but frequent monitoring is needed to follow the evolution of insecticide resistance.

## 1. Introduction

Since 2000, substantial progress has been made in fighting malaria. WHO estimates that between 2000 and 2015, malaria case incidence was reduced by 41% and malaria mortality rates by 62% [1, 2]. In 2016, there was an estimate of 216 million episodes of malaria and about 445,000 deaths globally, two-thirds of which were among children. Among the malaria-endemic countries in the WHO region of the Americas, Colombia contributed 15.3% of the total number of cases in 2016 and, in the last two years (2015-2016), the number of cases reported have doubled despite earlier reductions [3]. In relation to mosquito vectors,* Anopheles albimanus*,* An. darlingi, *and* An. nuneztovari s.l. *are widely distributed and are considered the primary vectors responsible for malaria transmission in most regions of the country [4].

In Colombia, the organochlorine insecticide DDT was intensively used for malaria control from 1947 until 1994 when its use was prohibited [5, 6]; since then, malaria vector control is supported mainly in the use of organophosphates and pyrethroid insecticides in indoor residual spraying (IRS) and pyrethroids in treated mosquito-nets (ITNs) in both impregnated nets and long lasting insecticidal nets (LLINs). Currently, the main insecticides used in Colombia for IRS are deltamethrin, lambda-cyhalothrin, and fenitrothion and, for ITNs, deltamethrin and alpha-cypermethrin are used. WHO has confirmed that prolonged use of insecticides has led to the development of resistance in malaria vectors against different insecticides used in public health. According to the 2017 World Malaria Report, 80% (61/76) of countries that provided surveillance data reported resistance to at least one insecticide class in one malaria vector from one collection site, and 65% (50/76) reported resistance to two or more insecticide classes [3].

Resistance monitoring in Colombia is done following WHO and CDC protocols, reporting insecticide resistance to pyrethroids, organophosphates, carbamates, and DDT, in the three primary malaria vectors [7–14]. According to current WHO resistance criteria (mortality rate <90%: resistance is confirmed) [1, 2], Fonseca-González [8] and INS (2015) reported deltamethrin, lambda-cyhalothrin, malathion, and DDT resistance in* An. albimanus* populations collected in the states of Antioquia, Valle del Cauca and Chocó. Studies by Fonseca-González et al. 2009b [10] and INS 2015 [12] documented insecticide resistance to lambda-cyhalothrin, deltamethrin, malathion, fenitrothion, and DDT in* An. nuneztovari s.l. *populations collected in Antioquia, Córdoba, Chocó, and Norte de Santander.* An. darlingi* collected in Antioquia, Chocó, Putumayo and Santander showed resistance to alpha-cypermethrin, deltamethrin, lambda-cyhalothrin, permethrin, fenitrothion, propoxur, and DDT [7, 9, 11, 12].

The Insecticide Resistance Surveillance Network (IRSN) in Colombia is led by the National Institute of Health and departmental entomology units, universities, research centers, and the Ministry of Health take part of this network. Work by IRSN has described the frequency, geographic distribution and in some cases the mechanisms responsible for the observed resistance. These data have been useful in decision-making. For example, when resistance to pyrethroids and DDT was identified in* An. darlingi* from Chocó in 2005-2006, IRS was promptly changed to organophosphate insecticide fenitrothion, to which no resistance had been detected [15].

Resistance frequency data obtained using the discriminating concentrations do not necessarily translate into efficacy rates in the field, data which is crucial to decisions required to deploy public health pesticides strategies [1, 2]. Consequently, WHO and CDC recently suggested that resistance phenotypes detected using the discriminating concentrations should be further assessed for their potential operational significance using methods developed to assess the resistance intensity in the same target vector population [1, 2, 16]. Furthermore, the data can be used to compare resistance levels between populations, monitor resistance evolution, and make operational decisions [1, 2]. To the best of our knowledge, there are no records evaluating resistance intensity in populations of resistant malaria vectors in the Americas.

Little is known about the mechanisms responsible for the resistance observed in these populations. Fonseca-González et al. [10] partially attributed the resistance to deltamethrin and lambda-cyhalothrin, observed in* An. nuneztovari s.l. *populations from Chocó and Norte de Santander, to the high mixed-function oxidase levels and the high levels of acetyl cholinesterase activity with the resistance to malathion in* An. nuneztovari s.l.* population of Antioquia, whereas for* An. darlingi* elevated levels of mixed-function oxidases and nonspecific esterases were associated with low mortalities against lambda-cyhalothrin and DDT from a population in Chocó [9]. Finally, a recent study developed by the National Institute of Health in the population of* Anopheles darlingi* from Tagachi, Quibdó-Chocó resistant to pyrethroids, found no evidence of the existence of mutations type* kdr* in the site L1014F [17].

Insecticide resistance is a problem of great concern and it needs to be monitored in order to maintain the efficacy of vector control operations in the field, even more when the countries in the Americas region have decided to achieve in 2020 the goal of reducing malaria morbidity by 40% or more, taking as a base the official figures of 2015 [18].

In this study, we describe the resistance levels and intensity of previously detected resistant populations among primary malaria vectors from the most malaria-endemic areas in Colombia.

## 2. Methods

### 2.1. Study Area

**Figure 1:**
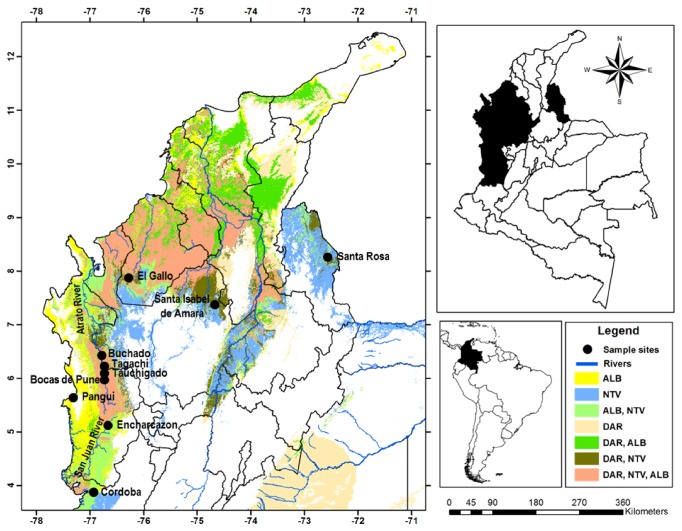
Field sites in Colombia. Distribution of the three primary* Anopheles* species represented by colors (ALB:* Anopheles albimanus*, NTV:* Anopheles nuneztovari s.l.*, and DAR:* Anopheles darlingi*). Black dots indicate sampling sites in the five states. These states are shown in black in the map of Colombia on the upper right panel. Colombia's location is shown in black in the South America map in the lower right panel.

The study was carried out in 10 localities of five states in Colombia: (Antioquia, Córdoba, Chocó, Norte de Santander, and Valle del Cauca) (Figure 1), areas with the highest burden of malaria in the country [5]. The study sites were chosen to encompass a range of primary malaria vector distribution, taking into account the previous results regarding pyrethroids and DDT resistance and biochemical mechanisms, rates of malaria incidence, easy access by land or water, safety, and public health priority in terms of resistance monitoring given the history of insecticide use.

To evaluate geographic range of previously reported resistance along the Atrato river in Antioquia and Chocó [7], we evaluated three locations around Tagachi separated by 17 to 30 kilometers.

### 2.2. Collection of Anopheles Mosquitoes

Anopheles were captured using human landing catches following the WHO standard recommendations of biosecurity to minimize the risk of malaria transmission in field technicians [19]. Collections were completed outdoors between 18:00 and 23:00 hours. All mosquitoes were identified morphologically in the field using taxonomic keys available for Colombia [20]. Adult female mosquitoes were caught directly in the field.

### 2.3. Biological Test for Determining Insecticide Susceptibility

Bioassays were performed between May and November 2016 and in August 2017, on* An. nuneztovari s.l.*,* An. darlingi*, and* An. albimanus*. The bioassays were carried out in each locality following the standard methodology of CDC Bottle Bioassay [21]. The method involved gently aspirating batches of 15–25 mosquitoes into BOECO GERMANY bottles with 250 ml capacity coated with the discriminating concentrations of the insecticides of interest to reach 100 mosquitoes (5-7 replicates with tests performed over one or more than one day) and two or more negative control bottles coated with acetone only. The number of dead or alive mosquitoes was recorded at fifteen-minute time-intervals until diagnostic time was reached. Temperature and humidity were recorded during exposure period and mortality rate scored at diagnostic time. After the diagnostic time exposure was reached in each bottle, surviving mosquitoes were transferred to a plastic container impregnated with triethylamine plaster to sacrifice them and stored separately from those that died postexposure. Taxonomic determination of all specimens was performed postbioassay due to presence of high* Anopheles *diversity. The solutions were prepared and the bottles coated according to the CDC protocol [21].

The insecticides, doses, and diagnostic times tested were for deltamethrin 12,5 *μ*g/bottle/30 minutes; lambda-cyhalothrin 12,5 *μ*g/bottle/30 minutes; alpha-cypermethrin 12,5 *μ*g/bottle/30 minutes; permethrin 21,5 *μ*g/bottle/30 minutes and DDT 100 *μ*g/bottle/30 minutes. Previous resistance reports and their use for IRS or LLINs by the National Malaria Control program justified the choice of these insecticides. Quality control of each insecticide solution was performed at the National Institute of Health using the* An. albimanus* susceptible strain Cartagena. Discriminating concentrations and diagnostic time applied in the present study were the recommended by CDC [21]. When insufficient numbers of mosquitoes were collected from sites to test all insecticides, insecticides were prioritized by history of resistance and selection pressure in the last five years.

### 2.4. Biological Test to Determine the Insecticide Resistance Intensity

When the mortalities in field populations of* Anopheles darlingi*,* An. albimanus* and* An. nuneztovari s.l.* were below 98%, intensity assays were performed to quantify the intensity of resistance, following the recommendations of WHO and CDC [1, 2, 16]. Briefly, bottles were treated with 2, 5, and 10 times the diagnostic dose of insecticide. The diagnostic time was not altered.

### 2.5. Data Analyses

The bioassay results were corrected using the abbott formula when the control mortality was between 5 and 15% [22]. Mortality percentages and confidence limits (95%) were calculated for each insecticide in each locality and its susceptibility status or intensity of resistance were defined according to the CDC criteria. In bioassays with diagnostic dose, resistant populations were defined as displaying less than 90% mortality, possibly resistant populations between 90–98% mortality, and susceptible ones greater than 98% [23]. In the intensity assays, mortality of 98-100% at the 2X, 5X, or 10X concentrations indicates low, moderate, or high resistance intensity, respectively.

All tests were conducted in the field at temperatures ranging from 16.5°C to 34.1°C with relative humidity ranging from 57% to 90% (Tables 1, 2, and 3).

### 2.6. Ethical Considerations

Ethics approval from the ethical committee of the National Health Institute of Colombia was obtained. Writen informed consent was obtained from all the participants CTIN 2-2016, act No. 9 of May 20, 2016.

## 3. Results

### 3.1. Insecticide Susceptibility Assays

Tables 1, 2, and 3 show each wild* Anopheles *spp. and insecticide from each field site with the corresponding mortality rate, 95% confidence interval and resistance status. Mortality ranged from 3 to 10% in the control groups except in Pangui, where the mortality averaged 14% against alpha-cypermethrin and lambda-cyhalothrin.

*An. albimanus* showed susceptibility to pyrethroids, although for lambda-cyhalothrin the mortality was lower than 98% which is then interpreted as possible resistance. This species showed resistance to DDT.

*An. darlingi* showed differential susceptibility status to pyrethriods and DDT. In the northern locality, Buchadó, this species was susceptible to all insecticides tested. However, in the southern localities by the Atrato river, possible resistance and confirmed resistance are shown to DDT as well as pyretroids (Table 2), except in the locality of San Francisco de Tauchigado, where this species was susceptible to all tested insecticides.

The species* An. nuneztovari s.l.* was susceptible in most of the tested localities to DDT and pyrethroids, and only in two localities possible resistance was found. In Cordoba (municipality of Buenaventura), Pacific Coast region, possible resistance to DDT and deltamethrin was found, as well as in Santa Rosa (municipality of Zulia), Estern region, and also mortalities between 90% and 98% were found to DDT (Table 3).

### 3.2. Insecticide Resistance Intensity

Resistance intensity was evaluated in the populations defined as resistant or possibly resistant according to the insecticide susceptibility assay. In all cases, for the three species tested, specimens exposed to 2X the discriminating concentrations showed 100% mortality indicating low intensity of resistance; therefore, it was not necessary to perform assays at 5 and 10X the diagnostic concentrations (Table 4).

## 4. Discussion

This study provides evidence that key malaria vectors in Colombia,* An. darlingi*,* An. albimanus*, and* An. nuneztovari s.l.,* were resistant to pyrethroids and DDT. This resistance was highly localized. Pyrethroids resistance in these same vectors was of low intensity, as 100% mortality was observed when exposed to 2X the discriminating insecticide concentrations. However, these results should be interpreted cautiously as the number of specimens evaluated by species and insecticide were all lower than 40 mosquitoes and 95% confidence intervals ranged from low to high intensity insecticide resistance.

This is the first study that aimed to follow up on previous reports of insecticide resistance and to evaluate resistance intensity. We have classified 10 areas and three vector species regarding the need to closely and routinely monitor insecticide susceptibility, i.e., sentinel sites. The evidence of persistent insecticide resistance implies that at least annual monitoring is necessary in these localities, while the program is distributing LLINs as the primary malaria vector control intervention. Given that IRS is also available and is performed with organophosphates and pyrethroids in the Pacific coast, the alternation of both LLINs and IRS with organophosphates could be a strategy to slow down the possible impact of pyrethroids, as recommended by WHO [15].

We observed changes in resistance compared to results previously obtained by the IRSN [7, 8, 12, 14]. The populations of* An. darlingi* from Tagachi and Bocas de Pune and the poulation of* An. albimanus* from Pangui continue to present results compatible with resistance to pyrethroids and DDT, although the percentages of mortality observed during this study were much higher (80.1%-97.8%) than those reported in previous studies (20.4 and 89%) [7, 8, 12]. These differences in mortality results may be due to the different genotype frequencies associated with the involved resistance mechanisms in the mosquitoes tested now compared with the mosquitoes tested in the past, as it has been found with* kdr* mutation in* An. gambiae* [24–26]. Alternatively, these higher mortality rates could be due to changes in selection pressure with insecticides. We find the population of* An. nuneztovari s.l.* from El Zulia to show resistance only to DDT in contrast with results found 11 years ago in this site, where resistance to lambda-cyhalothrin, fenithrotion, deltamethrin, and DDT was detected [10]. These changes towards susceptibility in this population could be explained by a decrease in the selective pressure with insecticides for malaria control, given the 88% reduction in the incidence of cases that has occurred in the last ten years [27, 28]. Another possible explanation is a decrease in the selective pressure due to lowered use of agricultural insecticides, given that the main economic activity in this area changed from rice crop to palm cultivation, a crop which is less pressured with insecticides due to lower pests' burden [29].

Additionally, we are the first to describe resistance data in populations of* An. nuneztovari s.l.* from Santa Isabel de Amará, Gallo, and Cordoba. The first two were susceptible to pyrethroids despite their wide use by the control program in indoor residual spraying with deltamethrin and lambda-cyhalothrin and LLINs with deltamethrin and alpha-cypermethrin. In Cordoba, we observed 94% and 95% mortality to DDT and deltamethrin, respectively, indicating that this malaria vector population should be carefully monitored as a priority in this state. This is of particular importance as this locality has been exerting continuous selective pressure with the scale-up with LLINs impregnated with alpha-cypermethrin and deltamethrin and IRS with deltamethrin.

The measurement of the relationship between insecticide resistance intensity and operational level control strategy failure has only recently been recommended [1, 2]. IRS failure has been observed when the levels of resistance intensity of the local vector populations are high [30]. This is less clear when using LLINs, attributed by some to the personal protection provided by the nets despite high level of vector resistance [31, 32]. WHO suggests interpreting resistance intensity assays as such that less than 98% mortality in mosquitoes exposed to 5 times and especially 10 times the discriminant concentration may indicate that control measures are ineffective in the field, indicating the urgency of developing adequate strategies for insecticide resistance management. According to the results found in this study, a low resistance intensity was observed among all tested species and insecticides pairs. It is probable that the low intensity of resistance detected does not yet pose a threat to malaria control in* An. darlingi* populations from Bocas de Pune, Tagachi and Encharcazon, in the population of* An albimanus* from Pangui, and in the population of* An. nuneztovari s.l.* from Cordoba. Nevertheless, given that Colombian vectorial control is based on distribution of LLINs (deltametrina or alpha-cypermethrin) and IRS (fenitrothion), we recommend at least annual resistance intensity surveillance for these three insecticides to develop timely strategies. This can prevent or delay increases in the intensity of resistance that may affect the operative control.

The evaluations of susceptibility made in the localities along the Atrato river, Bocas del Pune, Tauchigado, Tagachi, and Buchadó (<30 km distribution) demonstrated that resistance can be extremely localized, likely associated with variable insecticide use although it is difficult to determine given that there is no system that routinely reports these data. This localized variation highlights the importance of adapting local strategies for insecticide resistance management. Resistance to DDT, lambda-cyalothrin, deltamethrin, and permethrin was previously observed along the Atrato river in the locality of Amé-Beté [7, 9]. When pyrethroid resistance was detected, a recommendation was made to treat outbreaks and epidemics in this area with IRS with organophosphate fenitrothion instead of lambda-cyhalothrin and deltamethrin [33]. However, to date in Tagachi, both, organophosphates and pyrethroids are used in IRS and pyrethroids are used in LLINs. This can result in increased insecticide resistance to selective pressure. We could not follow up on this population in Amé-Beté during this study as this locality disappeared (personal communication JDP).

Despite the fact that DDT has not been used in Colombia since 1994, populations still remain resistant to DDT. It is possible that a genetic effect is still present in the population or that the subsequent and continuous pressures with pyrethroids are maintaining this cross-resistance. However, little is known about the mechanisms responsible for the resistance observed in these populations.

The taxonomic confirmation of the specimens used for insecticide resistance tests is necessary given the high diversity of species and the presence of species complexes.* An. nuneztovari s.l.*, for example, is considered a complex formed by at least three species [34–36]. Nevertheless, despite the high intraspecific morphological variation observed in specimens collected throughout its range of distribution in Colombia, the evidence indicated that* An. nuneztovari s.l.* constitutes a unique species in Colombia [37–40], which corresponds to* An. nuneztovari s.s.* [41]. In this study, although molecular determinations were not performed, we suppose that* An. nuneztovari* s.s was the species tested. The results with this species suggest possible resistance to the insecticide DDT in Santa Rosa and to the insecticides deltametrina and DDT in Cordoba. Furthermore, these localities have been exerting continuous selection pressure with the scale-up in coverage with LLINs treated with alpha-cypermethrin and deltamethrin and IRS with deltamethrin, lambda-cyhalotrin, and fenitrothion. Therefore, we recommend prioritizing in these states the careful monitoring of this malaria vector population.

Despite efforts form the Ministry of Health, National Institute of Health and the support of PAHO/WHO to increase awareness and capacity building for a Colombian Insecticide Resistance Surveillance Network, data on the prevalence of resistance in malaria vectors is scarce and geographically limited. Research may be limited by logistical difficulties in accessing relevant malaria-endemic areas, or high species diversity.

To highlight this, we found changes in species composition in previous evaluated localities. This was the case of* An. darlingi* in the locality of Encharcazon, Rio Iro - Choco, where before this species was not present and the predominant vector was* An. nuneztovari s.l.* [10]. The presence of* An. darlingi* resistant to DDT in this area was also unexpected. According to the historical records of distribution [20] and personal communications with technicians who worked for the malaria eradication service,* An. darlingi* was not previously present along the San Juan River. Therefore, it is likely that this resistant population comes from a population of resistant* An. darlingi* located along the Atrato river. However, there is no evidence of whether* An. darlingi* is replacing* An. nuneztovari s.l.* in this area or if these two species are exhibiting seasonality. This information is critical to define periods of resistance assessments, allowing for adjustments according to species of interest and the epidemiology of the disease given that both* An. nuneztovari s.l.* and* An. darlingi* are primary vectors of malaria in the country.

This is the first time that resistance intensity has been measured in Colombia and to our knowledge in the Americas. This information is useful for the local decision makers. Future priorities are to (1) strengthen the existing National Surveillance Insecticide Resistance Network and (2) to follow up on identified priority sentinel sites to obtain information on the susceptibility status of the main vectors to insecticides and intensity of resistance over time.

## 5. Conclusion

The results presented here demonstrate that highly focalized and low-intensity insecticide resistance has been maintained in some populations of the primary malaria vectors in Colombia. This may not pose a threat to malaria control yet. In general* An. darlingi*,* An. nuneztovari s.s.*, and* An. albimanus* were susceptible to three of the insecticides used in the country for malaria control, the pyrethroids: deltamethrin, lambda-cyhalothrin, and alpha-cypermethrin. In localities where resistance or possible resistance has been detected, routine surveillance is necessary to evaluate temporary changes. This will guide efficient use of vector control tools. It can also prevent unmanageable resistance levels that potentially compromise efficiency of malaria vectors control measures used in the field.

## Figures and Tables

**Table 1:** Average mortality rate, 95% confidence interval, and corresponding resistance interpretation (resistant (< 90% mortality), possibly resistant (90–98%), and susceptible (>98%)) among wild *An. albimanus* assayed via CDC bottle bioassays with discriminating concentrations.

**Locality, Municipality** **(State)**	**Temperature** °**C**	**Relative humidity** %	**Insecticide** **(concentration)**	**n**	%** mortality** **(95**%** CI)**	**CDC Susceptibility status**
Pangui, Nuqui (Choco)	24.29 - 29.7	81 - >90	Alpha-cypermethrin (12.5 ug/ml)	103	**100** (96.4 - 100)	Susceptible
			Deltamethrin (12.5 ug/ml)	109	**100** (96.6 - 100)	Susceptible
			Lambda-cyhalothrin (12.5 ug/ml)	109	**97.87** (95.16 - 100)	Possibly resistant
			Permethrin (21.5 ug/ml)	97	**100** (96.19 - 100)	Susceptible
			DDT (100 ug/ml)	101	**92.49** (87.35 - 97.63)	Possibly resistant

**Table 2:** Average mortality rate, 95% confidence interval, and corresponding resistance interpretation (resistant (< 90% mortality), possibly resistant (90–98%), and susceptible (>98%)) among wild *An. darlingi* assayed via CDC bottle bioassays with discriminating concentrations.

**Locality, Municipality** **(State)**	**Temperature** °**C**	**Relative humidity** %	**Insecticide** **(concentration)**	**n**	%** mortality** **(95**%** CI)**	**CDC Susceptibility status**
Buchado, Vigia del Fuerte (Antioquia)	24.1 - 27.8	68 - 83	Alpha-cypermethrin (12.5 ug/ml)	100	**100** (96.3 - 100)	Susceptible
			Deltamethrin (12.5 ug/ml)	164	**100** (97.71 - 100)	Susceptible
			Lambda-cyhalothrin (12.5 ug/ml)	101	**100** (96.34 - 100)	Susceptible
			Permethrin (21.5 ug/ml)	106	**100** (96.5 - 100)	Susceptible
			DDT (100 ug/ml)	101	**100** (96.34 - 100)	Susceptible

Bocas de Pune, Medio Atrato (Choco)	25.6 - 27.9	87 - >90	DDT (100 ug/ml)	112	**90.7** (85.32 - 96.08)	Possibly resistant

San Francisco de Tauchigado, Medio Atrato (Choco)	24.5 - 26.1	>90	Alpha-cypermethrin (12.5 ug/ml)	110	**100** (96,63 - 100)	Susceptible
			Deltamethrin (12.5 ug/ml)	110	**100** (96.63 - 100)	Susceptible
			Lambda-cyhalothrin (12.5 ug/ml)	112	**100** (96.68 - 100)	Susceptible
			Permethrin (21.5 ug/ml)	106	**100** (96.5 - 100)	Susceptible
			DDT (100 ug/ml)	105	**100** (96.47 - 100)	Susceptible

Tagachi, Quibdo (Choco)	25.5 - 33.6	65 - >90	Alpha-cypermethrin (12.5 ug/ml)	101	**100** (96.34 - 100)	Susceptible
			Deltamethrin (12.5 ug/ml)	107	**95.16** (91.09 - 99.23)	Possibly resistant
			Lambda-cyhalothrin (12.5 ug/ml)	104	**96.92** (93.59 - 100)	Possibly resistant
			Permethrin (21.5 ug/ml)	109	**95.93** (92.23 - 99.64)	Possibly resistant
			DDT (100 ug/ml)	99	**80.13** (72.27 - 87.99)	Resistant

Encharcazon, Rio Iro (Choco)	27.5 - 34.1	57 - 84	Deltamethrin (12.5 ug/ml)	113	**100** (95.86 - 100)	Susceptible
			DDT (100 ug/ml)	93	**95.7** (89.46 - 98.31)	Possibly resistant

**Table 3:** Average mortality rate, 95% confidence interval, and corresponding resistance interpretation (resistant (< 90% mortality), possibly resistant (90–98%), and susceptible (>98%)) among wild *An. nuneztovari s.l.* assayed via CDC bottle bioassays with discriminating concentrations.

**Locality, Municipality** **(State)**	**Temperature** °**C**	**Relative humidity** %	**Insecticide** **(concentration)**	**n**	%** mortality** **(95**%** CI)**	**CDC Susceptibility status**
Santa Isabel de Amara, Segovia (Antioquia)	25.6 - 31.3	59 - 84	Alpha-cypermethrin (12.5 ug/ml)	98	**100** (96.23 - 100)	Susceptible
			Deltamethrin (12.5 ug/ml)	113	**100** (96.71 - 100)	Susceptible

Santa Rosa, El Zulia (Norte de Santander)	16.5 - 22.8	<60	Alpha-cypermethrin (12.5 ug/ml)	119	**100** (96.87 - 100)	Susceptible
			Deltamethrin (12.5 ug/ml)	103	**100** (96.4 - 100)	Susceptible
			Lambda-cyhalothrin (12.5 ug/ml)	106	**100** (96.5 - 100)	Susceptible
			DDT (100 ug/ml)	124	**97.43** (94.65 - 100)	Possibly resistant

Cordoba, Buenaventura (Valle del Cauca)	25.7 - 27.5	78 - >90	Alpha-cypermethrin (12.5 ug/ml)	112	**100** (96.68 - 100)	Susceptible
			Deltamethrin (12.5 ug/ml)	107	**94.95** (92.83 - 97.06)	Possibly resistant
			Lambda-cyhalothrin (12.5 ug/ml)	102	**100** (96.37 - 100)	Susceptible
			Permethrin (21.5 ug/ml)	101	**100** (96.34 - 100)	Susceptible
			DDT (100 ug/ml)	100	**94** (87.52 - 97.22)	Possibly resistant

Gallo, Tierralta (Cordoba)	24.2 - 29.2	78 - 94	Alpha-cypermethrin (12.5 ug/ml)	174	**100** (97.84 - 100)	Susceptible
			Deltamethrin (12.5 ug/ml)	146	**100** (97.44 - 100)	Susceptible
			Lambda-cyhalothrin (12.5 ug/ml)	152	**100** (97.54 - 100)	Susceptible
			Permethrin (21.5 ug/ml)	141	**100** (97.35 - 100)	Susceptible
			DDT (100 ug/ml)	120	**100** (96.9 - 100)	Susceptible

**Table 4:** Average mortality rates, 95% confidence interval, and corresponding resistance intensity interpretation among wild *Anopheles *spp. assayed via CDC bottle intensity bioassays with 2x discriminating concentrations.

***Anopheles* specie**	**Locality, Municipality** **(State)**	**Temperature** °**C**	**Relative humidity** %	**Insecticide** **(concentration)**	**n**	%** mortality** **(95**%** CI)**	**Intensity of resistance**
*An. albimanus*	Pangui, Nuqui (Choco)	24.29 - 29.7	81 - >90	DDT (200 ug/ml)	21	100 (84.54 - 100)	Low intensity

*An. darlingi*	Bocas de Pune, Medio Atrato (Choco)	25.6 - 27.9	87 - >90	DDT (200 ug/ml)	38	100 (90,82 - 100)	Low intensity
	Tagachi, Quibdo (Choco)	25.5 - 33.6	65 - >90	Deltamethrin (25 ug/ml)	30	100 (88.65 - 100)	Low intensity
				Lambda-cyhalothrin (25 ug/ml)	25	100 (86.68 - 100)	Low intensity
				Permethrin (43 ug/ml)	31	100 (88.97 - 100)	Low intensity
				DDT (200 ug/ml)	30	100 (88.65 - 100)	Low intensity

*An. nuneztovari s.l.*	Cordoba, Buenaventura (Valle del Cauca)	25.7 - 27.5	78 - >90	Deltamethrin (25 ug/ml)	28	100 (87.94 - 100)	Low intensity
				DDT (200 ug/ml)	25	100 (86.68 - 100)	Low intensity

## Data Availability

The data used to support the findings of this study are available from the corresponding author upon request.
